# Modification of *PARP4*, *XRCC3*, and *RAD51* Gene Polymorphisms on the Relation between Bisphenol A Exposure and Liver Abnormality

**DOI:** 10.3390/ijerph17082794

**Published:** 2020-04-17

**Authors:** Jin Hee Kim, Yun-Chul Hong

**Affiliations:** 1 Department of Integrative Bioscience & Biotechnology, Sejong University, 209 Neungdong-ro, Gwangjin-gu, Seoul 05006, Korea; 2 Department of Preventive Medicine, Seoul National University College of Medicine, 28 Yongon-dong, Chongno-gu, Seoul 03080, Korea; ychong1@snu.ac.kr; 3 Institute of Environmental Medicine, Seoul National University Medical Research Center, 28 Yongon-dong, Chongno-gu, Seoul 03080, Korea

**Keywords:** bisphenol A, liver abnormality, repair gene polymorphism

## Abstract

Repair genes may play critical roles in the relationships between environmental exposure and health outcomes. However, no evidence is available about the effect of repair gene polymorphisms on the relationship between bisphenol A (BPA) exposure and liver abnormality. Therefore, we evaluated the effect of nine genotyped polymorphisms in three repair genes, *poly(ADP-ribose) polymerase family member 4* (*PARP4*), *X-ray repair cross complementing 3* (*XRCC3*), and *RAD51 recombinase* (*RAD51*), on the relationship between BPA exposure and liver abnormality using repeated measures data for an elderly population. A significant association between BPA levels and liver abnormality was found only in elders with the *PARP4* G-C-G haplotype, *XRCC3* G-A-G haplotype, or *RAD51* T-A-A haplotype (odds ratio (OR) = 2.16 and *p* = 0.0014 for *PARP4*; OR = 1.57 and *p* = 0.0249 for *XRCC3*; OR = 1.43 and *p* = 0.0422 for *RAD51*). Particularly, *PARP4* and *XRCC3* showed significant interactions with BPA exposure in relation to liver abnormality (*p* < 0.05 for both genes). These results indicate that *PARP4*, *XRCC3*, and *RAD51* gene polymorphisms have modification effects on the relationship between BPA exposure and liver abnormality.

## 1. Introduction

Bisphenol A (BPA) is one of the highest volume chemicals produced worldwide [1]. BPA production is also high in Korea based on emissions of > 17 tons every year in the workplace, meaning that BPA makes up 38.7% of the total potential endocrine disruptors that are released [2]. Humans are easily exposed to BPA because of its ubiquitous use in common consumer products, including beverage cans [3,4,5]. Because of its endocrine-disrupting properties in the body, it can induce various adverse sexual and non-sexual health outcomes [5,6,7,8,9,10,11]. 

Several studies have suggested that BPA may affect liver toxicity. An experimental study on BPA-treated rats showed marked liver defects resulting from BPA exposure, with elevations in three liver enzymes, aspartate alanine aminotransferase (ALT), aminotransferase (AST), and lactate dehydrogenase (LDH) [9]. Moreover, an American epidemiological study found a significantly positive association between BPA exposure and increases in three liver enzymes, alkaline phosphatase (ALP), γ-glutamyl transferase (γ-GTP), and LDH [10]. Liver abnormalities assessed using these liver enzyme levels are noteworthy due to their correlations with morbidity and mortality [12]. In a study assessing the risks of morbidity and mortality associated with liver abnormality using the National Health and Nutrition Examination Survey (NHANES) data, elevated ALT levels > 30 U/L for men and >19 U/L for women increased 11.2 times in liver-related mortality and 3.3 times in diabetes-related mortality [12]. This trend was found in a Korean study as well, with considerable increases (8–9.5 times) in the relative risk of liver-related deaths in relation to AST and ALT increases [13]. The frequency of liver abnormalities assessed using these liver enzyme levels was high in elderly populations, especially in female elders compared with other age groups, and abnormalities were even found in the male elders who did not drink alcohol [14]. The number of adults aged > 60 years is continuously increasing worldwide, as well as in Korea, and this number is expected to double between 2015 and 2050 [15].

Oxidative stress could be important in the relationship between BPA exposure and liver toxicity [9,16,17]. Because oxidative stress produced by BPA exposure can induce DNA damage, such as oxidized bases and strand breaks, BPA-associated oxidative stress could be important to the instability of organs, including the liver [18]. For this reason, a DNA repair mechanism may be important in order to maintain the genomic integrity of organs, including the liver [18]. Although several studies have supported the induction of oxidative stress by BPA exposure, an increase in liver toxicity through oxidative stress, and the induction of liver enzymes from damaged livers [19,20], there is limited evidence about the relationships between BPA exposure, oxidative stress, liver abnormality, and repair genes. 

There are two major types of repair mechanisms for DNA breaks—base excision repair (BER) for single-strand DNA breaks and homologous recombination repair and non-homologous end-joining for double-strand DNA breaks [21,22]. Poly(ADP-ribose) polymerase family member 4 (PARP4) is involved in the BER pathway in order to remove oxidized DNA bases [21]. X-ray repair cross-complementing 3 (XRCC3) is a member of the RAD51 recombinase (RAD51)-related protein family that participates in homologous recombination for stable chromosome maintenance, resulting in double-strand break repair [22]. RAD51 interacts directly with XRCC2, XRCC3, and breast cancer genes (*BRCA1* and *BRCA2*) to form a complex that is essential for double-strand break repair [22]. These three genes (*PARP4*, *XRCC3*, and *RAD51*) have been reported as playing vital repair-related roles in the protection against various diseases, including tumorigenesis [23,24,25,26,27,28,29]. Therefore, we genotyped *PARP4*, *XRCC3*, and *RAD51* polymorphisms, and evaluated the effect of these polymorphisms on the relationships between BPA exposure and oxidative stress and/or liver abnormalities in the elderly population.

## 2. Materials and Methods 

### 2.1. Study Population and Sampling

The present study was conducted to evaluate the effect of repair gene polymorphisms on the associations between BPA exposure and oxidative stress and liver abnormality, measured in repeatedly collected urine and blood samples. Briefly, a total of 560 elders aged 60 or over were included in the study. They visited a community welfare center in Seoul a maximum of five times for health checkups (twice in 2008, once in 2009, and twice in 2010) [30]. Urinary samples for BPA and malondialdehyde (MDA) measurements were collected at every visit and fasting blood samples for liver enzyme measurements and genotyping were collected three times (once per year) [30]. To test the relationship between BPA level and liver abnormality, we excluded six elders diagnosed with liver diseases, such as liver cancer, viral hepatitis, fatty liver disease or triglyceride (TG) > 800 mg/dL. To accurately measure BPA and MDA levels, we excluded seventeen elders diagnosed with repeated renal failure with BUN-creatinine ratios > 30. We also excluded 35 elders whose urine and blood were unavailable or who did not have DNA for genotyping. Finally, 502 elders were included in the analyses. The Institutional Review Board (IRB) at Seoul National University Hospital approved our study protocol (IRB no. H-0804-045-241) and each study participant provided written informed consent.

### 2.2. Urinary BPA, MDA, and Cotinine Levels

We measured urinary BPA levels using high-performance liquid chromatography (HPLC)–tandem mass spectrometry (MS) (Agilent 1200 for HPLC and Agilent 6410 Triple Quad LCMS for MS/MS) according to previously reported procedures [31,32]. Quality control was conducted following the Clinical and Laboratory Standards Institute guidelines with 99.7% accuracy, a 1.0–4.7 coefficient of variation for precision, and a 0.5–5.3 coefficient of variation for reproducibility. Urinary MDA levels were determined by thiobarbituric acid-reactive substance measures according to previously reported procedures [31,32]. The limit of detection (LOD) was 0.01 μg/L for BPA and 0.012 μmol/L for MDA. BPA levels less than the LOD were imputed as the LOD level divided by two, because our data were highly skewed [33], whereas all MDA levels were over the LOD level. We also measured urinary cotinine levels to monitor tobacco exposure using enzyme-linked immunosorbent assays [34]. Because the detection range for cotinine was 1–10,000 mg/L, cotinine levels out of the range were imputed as 0.5 mg/L for cotinine levels < 1 mg/L and 15,000 mg/L for cotinine levels >10,000 mg/L. To control for the different urinary excretion rates, creatinine-corrected BPA, MDA, and cotinine levels were used in all analyses. To measure urinary creatinine levels, a kinetic colorimetric assay was conducted using the CREA reagent (Roche, Indianapolis, Indiana, USA) on a Hitachi 7600 (Hitachi, Tokyo, Japan). 

### 2.3. Serum Liver Enzyme and Low-density Lipoprotein Cholesterol (LDL-C) Levels

ALT, AST, and γ-GTP are hepatocellular injury markers. The serum levels of the three enzymes were measured according to previously reported procedures [11] and those levels were used to detect liver abnormalities. Firstly, we set the upper limits of normal ALT, AST, and γ-GTP ranges in the aged population: 45 IU/L for ALT, 35 IU/L for AST, and 60 IU/L for γ-GTP. Secondly, if any one of the three liver enzymes were > 1.5 times the value, it was considered a clinically significant liver abnormality, which could be associated with increases in morbidity and mortality. A binary liver abnormality outcome (one vs. zero), calculated based on these criteria, was used as a dependent variable in the statistical analyses.

Since low-density lipoprotein cholesterol (LDL-C) levels are considered to affect liver abnormality [11], we adjusted the serum LDL-C level in our statistical models of the relationship between BPA exposure and liver abnormality. To determine serum LDL-C levels, we first measured the serum levels of total cholesterol (TC), high-density lipoprotein cholesterol (HDL-C), and TG using previously reported procedures [11]. Secondly, we calculated LDL-C levels using the TC, HDL-C, and TG levels according to the following equation: LDL-C (mg/dL) = TC (mg/dL)–HDL-C (mg/dL)–[TG (mg/dL)/5.0] [35]. Thirdly, if the calculated LDL-C level had a negative value, it was assigned as zero.

### 2.4. Air Pollutant Concentration and Meteorological Factors

Because particulate matter less than 10 μm (PM_10_) on lag day 2 and meteorological factors (outdoor temperature and dewpoint) on the day were associated with MDA levels in our previous study [32], we adjusted for PM_10_, outdoor temperature, and dewpoint in our models in order to evaluate their relationships with MDA. Data for PM_10_ measured at the monitoring center nearest to the residence of each subject was obtained from the Korean National Institute of Environmental Research [32]. The temperatures and dewpoints measured at the Songwol-dong monitoring center were obtained from the Korean Meteorological Administration [32]. 

### 2.5. Genotyping of PARP4, XRCC3, and RAD51, and Haplotype Estimation

To evaluate the effect of *PARP4*, *XRCC3*, and *RAD51* genes on the relationship between BPA exposure and outcomes, we selected target single nucleotide polymorphisms (SNPs) of *PARP4*, *XRCC3*, and *RAD51* genes, considering linkages among SNPs and proxy effects in other sites due to these linkages. Regardless of their known functionality, the nine SNPs (rs12863638, rs3783073, and rs2275660 for *PARP4*; rs861531, rs12432907, and rs861537 for *XRCC3*; rs2619681, rs2304579, and rs2412547 for *RAD51*) were tagged pairwise based on the Hardy–Weinberg equilibrium (HWE) > 0.05, r^2^ ≥ 0.8 (r^2^ ≥ 0.6 only for *PARP4*), a logarithm of the odds ≥ 3, missing < 10%, and a minor allele frequency > 0.1 for Han Chinese in Beijing and Japanese in Tokyo, using a Haploview 4.2 Tagger (version 3) and HapMap R2 data. 

Genomic DNA was extracted from each individual’s blood once, even though we collected blood samples a maximum of three times. Individual DNA samples were extracted using a QIAamp DNA Blood Mini Kit (Qiagen, Valencia, CA, USA) and nine *PARP4*, *XRCC3*, and *RAD51* gene polymorphisms were determined using the TaqMan fluorogenic 5′ nuclease assay (ABI, Foster City, CA, USA). The final volume of the polymerase chain reaction (PCR) was 5 μl, containing 10 ng of genomic DNA, 2.5 μl of TaqMan Universal PCR Master Mix, and 0.13 μl of 40X Assay Mix (Assay ID, C_9228399_10 for rs12863638, C_27515784_10 for rs3783073, C_15879320_10 for rs2275660, C_8901543_10 for rs861531, C_2983916_20 for rs12432907, C_2983919_10 for rs861537, C_26634294_20 for rs2619681, C_15975048_10 for rs2304579, and C_15794698_10 for rs2412547). Thermal cycling and fluorescent readings were conducted with negative controls, as conducted in a previously reported procedure [30]. Five percent of the samples were randomly chosen for repeat testing. We obtained identical results with 100% accuracy for the repeat genotyping.

### 2.6. Statistical Analysis 

The present study performed repeated measures of BPA, MDA, and liver enzymes for each individual. To evaluate the short-term effect of BPA level changes on MDA level and liver abnormality, we used mixed effect models for MDA and GLIMMIX models for liver abnormality. In both models, the BPA and MDA levels were urinary creatinine-corrected and then naturally log-transformed for their normalization. In the mixed effect model for MDA, estimated (β) values and 95% confidence intervals (CI) were obtained after adjustment for age (year), sex (male/female), body mass index (BMI, kg/m^2^), alcohol consumption status (yes/no), regular exercise (yes/no), cotinine level (mg/g-creatinine), PM_10_ on lag day 2 (µg/m^3^), and mean temperature (℃) and dewpoint (℃) on the day. In the GLIMMIX model, to calculate the odds ratio (OR) for liver abnormality, we used binary outcomes for liver abnormality. In the models, ORs and 95% CI were obtained after adjustment for age, sex, BMI, alcohol consumption status, regular exercise, cotinine levels, and LDL-C (mg/dL). The subject was treated as a random effect in both models.

The distributions of observed and expected genotype frequencies were compared using χ^2^-tests to identify whether each SNP site was on the HWE. Incomplete data with genotypes missing for at least one SNP on each gene were excluded in the estimation of individual haplotypes using the PHASE program (version 2.0.2). Pairwise linkage disequilibrium (LD) among the polymorphic sites for the *PARP4*, *XRCC3*, and *RAD51* genes was estimated as relative disequilibrium (D′) with Fisher’s exact test. We also estimated the relationship between BPA exposure and oxidative stress and liver abnormality by the genetic polymorphisms, haplotypes, and diplotypes of *PARP4*, *XRCC3*, and *RAD51*, using the same models and same covariates that were used in the estimation of the relationship between BPA exposure and oxidative stress and liver abnormality.

For statistical analyses, we used SAS version 9.4 (SAS Institute Inc., Cary, NC, USA) with a significance level of *p* < 0.05.

## 3. Results

### 3.1. Demographics

Our study participants included a total of 502 elders without liver disease or renal failure and with DNA analysis results. At the first visit, the participants were, on average, 70.8 years old and the number of males was 133 (26.5%) (Table 1). The mean visit number of the individuals was 3.3 (Table 1). The number of obese elders with BMI ≥ 25 was 224 (44.6%), and current smokers, current drinkers, and regular exercisers were 5.8%, 20.1%, and 60.6%, respectively (Table 1).

### 3.2. Distribution of Repeated Levels of BPA exposure, Oxidative Stress, and Liver Abnormality Markers

The distribution of the repeated measures of BPA, MDA, liver abnormality-related markers, and covariates affecting the relationships between BPA levels and levels of MDA or liver abnormality-related markers during the study period are shown in Table 2. BPA was detected in 1453 urine samples out of 1483 and the mean BPA level was 1.2 μg/L, whereas MDA was detected in all urine samples and the mean MDA level was 1.9 μmol/L (Table 2). The means of the creatinine-corrected BPA and MDA levels were 1.4 μg/g-creatinine with 4.3 of standard deviation (SD) and 0.5 μmol/g-creatinine with 0.4 of SD, respectively (Table 2). Nine hundred and fifty urine samples were detected in the range 1–10,000 mg/L for urinary cotinine, whereas 529 urine samples were under 1 mg/L and 10 urine samples were over 10,000 mg/L. The arithmetic mean levels of ALT, AST, and γ-GTP measured in 1002 sera were 20.0 IU/L, 21.9 IU/L, and 25.5 IU/L, respectively. Almost all liver enzyme levels (96.8%) were within the normal range for all three liver enzymes.

### 3.3. Information for PARP4, XRCC3, and RAD51 Gene Polymorphisms and HWE

The nine genotyped polymorphisms in the *PARP4*, *XRCC3*, and *RAD51* genes are listed in Table 3. The call rate was high, with a minimum of 98.6% for the nine polymorphisms (Table 3). The genotype frequencies for rs12863638, rs3783073, rs2275660, rs861531, rs12432907, rs861537, rs2619681, and rs2412547 were on the HWE, but those for rs2304579 were not on the HWE (*p* < 0.05 by χ^2^-test for rs2304579) (Table 3). 

### 3.4. Relations between BPA Exposure and Oxidative Stress and Liver Abnormality by Repair Gene Genotypes

The most common genotype was GT (46.0%) for rs12863638, CT (46.9%) for rs3783073, AG (44.4%) for rs2275660, GG (88.1%) for rs861531, AG (49.6%) for rs12432907, GA (46.4%) for rs861537, TT (49.0%) for rs2619681, AA (77.7%) for rs2304579, and AA (69.4%) for rs2412547 (Table 4). The BPA level was strongly associated with the MDA level (β = 0.10, 95% CI: 0.07–0.13, *p* < 0.0001) (Table 4). This association was found for most genotypes of *PARP4*, *XRCC3*, and *RAD51*, even though significance was not found in the small number of participants with minor alleles for rs2275660, rs861531, rs2304579, and rs2412547 (Table 4). However, in the evaluation of the relationship with liver abnormality by genetic polymorphisms of *PARP4*, *XRCC3*, and *RAD51*, high BPA exposure was significantly related to liver abnormality (adjusted OR = 1.46, 95% CI: 1.04–2.05, and *p* = 0.0308), particularly in participants with the CC genotype of rs3783073 (adjusted OR = 1.96, 95% CI: 1.18–3.26, and *p* = 0.0093), GG genotype of rs2275660 (adjusted OR = 2.63, 95% CI: 1.07–6.44, and *p* = 0.0354), GG genotype of rs861531 (adjusted OR = 1.48, 95% CI: 1.05–2.09, and *p* = 0.0250), AG genotype of rs12432907 (adjusted OR = 2.17, 95% CI: 1.24–3.78, and *p* = 0.0065), GA genotype of rs861537 (adjusted OR = 1.80, 95% CI: 1.02–3.20, and *p* = 0.0434), AA genotype of rs2304579 (adjusted OR = 1.65, 95% CI: 1.14–2.40, and *p* = 0.0083) or AA genotype of rs2412547 (adjusted OR = 1.79, 95% CI: 1.18–2.71, and *p* = 0.0067) (Table 5).

### 3.5. Relations between BPA Exposure and Oxidative Stress and Liver Abnormality by Repair Gene Diplotypes

When we estimated LDs among the polymorphic sites for *PARP4*, *XRCC3*, and *RAD51* genes, each polymorphic site was strongly linked with the other site in the same gene (rs12863638 and rs3783073, D′ = 0.95; rs3783073 and rs2275660, D′ = 0.98; rs861531 and rs12432907, D′ = 1; rs12432907 and rs861537, D′ = 1; rs2619681 and rs2304579, D′ = 1; rs2619681 and rs2412547, D′ = 1; rs2304579 and rs2412547, D′ = 0.99) even though strong linkage was not found between rs12863638 and rs2275660 for the *PARP4* gene and between rs861531 and rs861537 for the *XRCC3* gene (rs12863638 and rs2275660, D′ = 0.71; rs861531 and rs861537, D′ = 0.52) based on linkage with D′ of more than 0.8. Each polymorphism in the *PARP4*, *XRCC3*, and *RAD51* genes may act as a proxy of the other sites in the same gene because of their strong linkages. Therefore, we estimated the haplotypes that were composed of polymorphisms in each gene and evaluated the relationships between BPA exposure and oxidative stress and liver abnormality using their combined diplotypes (Table 4 and Table 5). First, we divided the elders into two groups with or without specific haplotypes for each gene, due to the small sample size of each haplotype. Then, the relationships between BPA exposure and oxidative stress and liver abnormality were evaluated in the two groups separately. In the analyses of the combined diplotypes of each gene, BPA exposure was associated with increased liver abnormality, particularly in elders with specific haplotypes for each gene (*PARP4*: adjusted OR = 2.16, 95% CI: 1.35–3.45, and *p* = 0.0014; *XRCC3*: adjusted OR = 1.57, 95% CI: 1.06–2.32, and *p* = 0.0249; *RAD51*: adjusted OR = 1.43, 95% CI: 1.01–2.01, and *p* = 0.0422) (Table 5). However, we did not find any difference in the association between BPA and MDA levels in elders with or without specific haplotypes for each gene, showing significant associations between BPA and MDA levels for all combined diplotypes of each gene (Table 4). 

### 3.6. Direct Effects of Repair Gene Polymorphisms on Liver Abnormality and Interactions with BPA Exposure

We also tested the effects of genetic polymorphisms and combined diplotypes on oxidative stress and liver abnormality (Table 6 and Table 7). No genotype or diplotype affected oxidative stress, regardless of whether we controlled for BPA exposure (Table 6). Nevertheless, an interaction between *XRCC3* rs861531 and BPA exposure was found to be significant for oxidative stress (*p* = 0.0445 among GG, GT, and TT genotypes; *p* = 0.0077 between GG genotype and GT or TT genotype) (Table 6). For liver abnormality, however, the *PARP4* rs2275660 GG genotype was risky compared with the AA genotype (adjusted OR = 3.3, 95% CI: 1.16–9.13, and *p* = 0.0387) (Table 7). This trend was found even after adjustment for BPA exposure (adjusted OR = 3.3, 95% CI: 1.16–9.27, and *p* = 0.0387) (Table 7). Furthermore, we also found a significant interaction between *PARP4* rs2275660 and BPA exposure in relation with liver abnormality (*p* = 0.0287 among AA, AG, and GG genotypes; *p* = 0.0085 between AA genotypes and AG or GG genotypes) (Table 7). This significant interaction with BPA exposure still remained for *PARP4*-combined diplotypes, with a risky effect of *PARP4* G-C-G haplotype including rs2275660 G allele (*p* = 0.0078) (Table 7). In addition, we also found significant interactions between *RAD51* rs2304579 or rs2412547 genotypes and BPA exposure for liver abnormality, despite the fact that *RAD51* rs2304579 and rs2412547 genotypes had no effect on liver abnormality (*p* = 0.0122 between AA genotype and AG or GG genotypes for rs2304579; *p* = 0.0241 among AA, AC, and CC genotypes for rs2412547; *p* = 0.0229 between AA genotype and AC or CC genotypes for rs2412547) (Table 7).

## 4. Discussion

The present study results show that the polymorphisms or combined diplotypes of repair genes *PARP4*, *XRCC3*, and *RAD51* modified the relationship between BPA exposure and liver abnormality, although none of the nine polymorphisms in the three genes modified the relationship between BPA and MDA levels. 

PARP4 is a protein involved in the removal of oxidized DNA bases. It has two important domains for its activation, a breast cancer 1 (BRCA1) protein C-terminus (BRCT) domain at the NH2 terminus and a major vault protein (MVP)-interacting domain at the COOH terminus [36,37,38]. BRCT repeats on PARP4 are known to bind phosphorylated DNA damage-sensing proteins, recruiting PARPs to DNA damage sites [21]. The MVP-interacting domain binds MVP, which responds to xenobiotic exposure and serves as a scaffolding protein in signaling and intracellular transport pathways, increasing malignancy in certain types of cancer, including colon cancer [24,39]. In particular, a previous study on PARP4 knockout mice indicated that PARP4 may directly or indirectly be involved in chemically induced tumorigenesis [24]. Because of this functional evidence for PARP4, its genetic polymorphisms have been studied in association with several diseases. In a study of 48–52-year-old male hepatocellular carcinoma patients diagnosed with chronic hepatitis B, *PARP4* variants were detected in > 10% of the whole-genome sequencing samples, with functional inhibitions of DNA BER and tumor cell apoptosis *in vivo* [36]. A genome-wide association study of 309 salivary gland carcinoma (SGC) patients and 535 controls without any carcinoma identified that common genetic variants in *PARP4* were associated with SGC development [28]. In 14 female cancer patients with both breast and thyroid primary malignancies, rare variations in *PARP4* (G496V and T1170I) were frequently detected with 5.2 ORs, and the knockdown of PARP4 with siRNA in an HCC1143 cell line significantly enhanced cell proliferation [40]. Furthermore, Kaplan–Meier analyses using public databases (GEO, EGA, and TCGA) showed poor progression-free and overall survivals (hazard ratios of 0.71 and 0.79, respectively) in a low PARP4-expressing group [40]. However, there was no evidence suggesting that *PARP4* gene modified the relationship between BPA exposure and liver toxicity, except for one report of frequent *PARP4* V458I mutations in a liver metastatic cohort [41].

XRCC3 repairs double-strand breaks [22]. A study on the association between *XRCC3* polymorphisms and bladder carcinoma examined the effect of *XRCC3* haplotypes composed of the polymorphisms (rs86153, rs861531, rs861537, rs861534, and rs1799794) on bladder cancer development, and the T-A haplotype with a T allele on rs861531 and an A allele on rs861537 showed protective effects for bladder cancer [29]. This result was consistent with the present study results indicating the risks associated with haplotypes with a G allele on rs861531 and a G allele on rs861537. However, in diplotype analyses of a Chinese case control study on non-small-cell lung cancer (NSCLC), an *XRCC3* rs1799794 A allele on haplotypes composed of rs861539–rs1799796–rs861537–rs1799794 was associated with an increased NSCLC risk regardless of the A or G alleles on rs861537, although subjects with rs861537 AA or AG genotypes showed increased ORs for NSCLC in genotype analyses [42]. This indicates that it is important to consider not only the effects of genotypes on outcomes, but also the effects of haplotypes or diplotypes, regardless of the disease. Furthermore, there are no reports related to liver diseases, except for a study of *XRCC* rs861539 and rs1799796 associated with tumor grade or survival in hepatocellular carcinoma patients [43].

RAD51 is also related to repairing double-strand breaks [22]. Several studies have shown that *RAD51* genetic polymorphisms may contribute to RAD51 expression differences among individuals, leading to the development of breast cancer [23,25,26,27]. However, the results for the association of *RAD51* genetic polymorphisms with cancer development have been contradictory [22,27,44,45,46]. Tulbah et al. [22] reported that the rs2619681 CC genotype or C allele in the *RAD51* gene was associated with increased breast carcinoma risks in Saudi females. However, no evidence has been reported for the effect of *RAD51* genetic polymorphisms on liver toxicity or abnormality. 

In the present study, we also explored the effects of repair gene polymorphisms and their combined diplotypes on oxidative stress and liver abnormality. Although direct effects of repair gene polymorphisms and combined diplotypes were not found for oxidative stress, a significant interaction between *XRCC3* rs861531 and BPA exposure was found for oxidative stress. However, we could not calculate the interactive effect of *XRCC3* rs861531 and BPA exposure on liver abnormality because there were no cases of liver abnormality in the GT or TT genotype strata, due to the relatively small number of GT or TT genotypes compared with GG genotypes. Furthermore, the direct effect of the *PARP4* rs2275660 GG genotype on liver abnormality was parallel to the relationship between BPA exposure and liver abnormality obtained from each genotype stratum, indicating that the GG genotype could directly affect liver abnormality, and also that the effect of BPA exposure could be more apparent for liver abnormality in this genotype stratum. In fact, the interaction between *PARP4* rs2275660 (or *PARP4* diplotypes, including this SNP) and BPA exposure was found to be significant for liver abnormality in our study. *RAD51* rs2304579 and rs2412547 possess somewhat similar effects to *PARP4* rs2275660. In other words, *RAD51* rs2304579 and rs2412547 showed interactive effects with BPA exposure for liver abnormality, with somewhat decreased ORs in rs2304579 AG or GG genotype and rs2412547 AC or CC genotype strata and apparent effects of BPA exposure in rs2304579 AA genotype and rs2412547 AA genotype strata. Based on the evidence that BPA exposure induces the expression of *BRCA1* and *BRCA2*, as well as *RAD51*, in human breast epithelial cells and in worms on late pachytene, a stage in which levels of this strand invasion/exchange double-strand break repair (DSBR) protein normally decrease [47,48], a significant interaction between the *RAD51* gene and BPA exposure found in our study could be plausible. However, we did not find any reports on the interactions between the repair genes examined in our study and BPA exposure.

In our previous study, for the effects of polymorphisms in oxidative stress-related genes (*COX2*, *EPHX1*, *HSP70-hom*, *PON1*, *eNOS*, *CAT*, *DRD2*, *SOD2*, and *MPO*) on the relationship between BPA exposure and the level of malondialdehyde (MDA) as an oxidative stress biomarker, we did not find any effects for the polymorphisms, even though we found a strong association between BPA exposure and MDA level [31]. However, in another previous study, the polymorphisms of four oxidative stress-related genes (*COX2*, *EPHX1*, *CAT*, and *SOD2*) affected the relationship between BPA exposure and liver abnormality [30]. Based on the two previous study results [30,31], we hypothesized that the reason why oxidative stress-related gene polymorphisms had no effect on the association between BPA exposure and MDA level may be because of the momentary effect of BPA, with a short biological half-life < 6 hours, on the MDA level, which occurred before the defense system of the body became active. This phenomenon, which occurred in our previous studies, was also observed in the analyses of repair gene polymorphisms in the present study.

When we calculated the AST/ALT ratio, 364 (36.3%), 611 (61.0%), and 27 (2.7%) samples were under one, between one and two, and over two, respectively (data not shown here). Among 27 samples with AST/ALT ratios over two, only one sample showed high γ-GTP levels, which we expected would indicate alcoholic liver disease, although we excluded all subjects with liver diseases [49]. However, the subject who provided this sample was a non-drinker, not an ex-drinker or current drinker. Furthermore, because AST/ALT ratios over 1 could be attributable to tissues other than liver as well, high AST/ALT ratios originating from other tissues, such as muscle, need to be considered in the future.

In addition, we evaluated whether temporal BPA exposure for each individual was sufficiently changeable in order to evaluate the effect of repair gene polymorphisms on the relationship between BPA exposure and oxidative stress and/or liver abnormality. In our evaluation of the temporal BPA exposure, the within-individual correlation coefficient for BPA ranged between 0.11 and 0.14 (data not shown here), indicating that within-individual variation was much larger than between-individual variation. This means that temporal BPA exposure for each individual was sufficiently changeable in our evaluation of the relationships between BPA exposure, oxidative stress, liver abnormality, and repair gene polymorphisms.

There were several strengths to the present study. Firstly, to the best of our knowledge, this is the first epidemiological study to investigate the role of repair gene polymorphisms between BPA exposure and liver abnormality. Secondly, the panel study design, with repeated measurements of BPA and liver abnormality-related markers for each participant, allowed for the evaluation of the short-term effects of temporal BPA exposure, minimizing any confounding effects that could be produced from basic personal characteristics, including chronic medical conditions. Nevertheless, the liver abnormality dependency on the repair gene polymorphisms should be investigated in future studies, because it may occur as a result of chronic BPA exposure on the liver. Our study also had some limitations. If age modifies the relationships among BPA exposure, oxidative stress, liver abnormality, and repair gene polymorphisms, our study results (obtained from elders aged 60 years or older) may not be generalizable to younger people. Therefore, we need to confirm our results in a younger population in the future.

## 5. Conclusions

In this study, we showed the modification of *PARP4*, *XRCC3*, and *RAD51* gene polymorphisms on the relationship between BPA exposure and liver abnormality. Our findings provide useful information to identify susceptible populations who are targets for public health interventions. Enhancing the repair capacity in susceptible populations, in addition to regulating BPA exposure, could potentially prevent liver diseases.

## Figures and Tables

**Table 1:** Demographic characteristics of participants at the first visit.

Characteristic	Total
No. of participants (%)	502 (100)
Mean age, years (min-max)	70.8 (60−87)
No. of males (%)	133 (26.5)
Visit no. (mean ± SD)	3.3 ± 1.4
Height [mean ± SD (cm)]	154.9 ± 7.6
Weight [mean ± SD (Kg)]	59.6 ± 9.0
BMI (kg/m^2^), no. (%)	
≥ 25	224 (44.6)
23 ~ < 25	154 (30.7)
< 23	124 (24.7)
No. of current smokers (%)	29 (5.8)
No. of current drinkers (%)	101 (20.1)
Regular exercise, no. of yes (%)	304 (60.6)

**Table 2:** Distribution of repeated levels of bisphenol A (BPA), oxidative stress or liver abnormality markers, and covariates affecting the relationship between BPA and each marker during the study period.

					Selected Percentiles		
Chemicals	Observation No.	LOD	No. <LOD or >LOD	Mean (SD)	25th	50th	75th
BPA (μg/L)	1483	0.01	30	1.2 (2.7)	0.4	0.7	1.2
BPA (μg/g-creatinine)	1482			1.4 (4.3)	0.4	0.7	1.4
MDA (μmol/L)	1495	0.012	0	1.9 (1.2)	1.1	1.7	2.4
MDA (μmol/g-creatinine)	1488			0.5 (0.4)	0.3	0.4	0.6
Cotinine (mg/L)	1489	1 or 10,000	529 for <1 and 10 for >10,000	271.7 (1556.2)	0.5	2.0	4.4
Cotinine (mg/g-creatinine)	1488			242.1 (1397.8)	0.9	2.1	4.6
Creatinine (mg/dL)	1492			106.0 (60.7)	64.8	94.7	134.0
TC (mg/dL)	1002			189.6 (37.0)	164.0	188.0	213.0
TG (mg/dL)	1000			137.5 (80.6)	85.0	118.0	168.0
HDL-C (mg/dL)	1000			51.3 (13.3)	42.0	50.0	59.0
LDL-C (mg/dL)	1000			110.9 (33.8)	87.4	108.8	133.1
ALT (IU/L)	1002			20.0 (9.8)	14.0	18.0	23.0
AST (IU/L)	1002			21.9 (8.4)	17.0	20.0	24.0
γ-GTP (IU/L)	1002			25.5 (20.8)	15.0	19.0	27.0
PM_10_ on lag day 2 (µg/m^3^)	1606			41.2 (23.4)	26.4	36.4	52.5
Temperature on the day (℃)	1652			16.8 (9.0)	9.8	18.0	24.9
Dewpoint on the day (℃)	1652			6.1 (10.8)	−2.0	7.7	15.3

The limit of detection (LOD) levels were 0.01 μg/L for BPA and 0.012 μmol/L for malondialdehyde (MDA). BPA concentrations under LOD were assigned as the LOD concentration divided by two, whereas all MDA levels were over the LOD level. Creatinine-corrected urinary BPA and MDA concentrations were used to control the different urinary excretion rates. Because the detection range for cotinine was 1–10,000 mg/L, cotinine concentrations under 1 mg/L and over 10,000 mg/L were assigned as 0.5 mg/L and 15,000 mg/L, respectively. Creatinine-corrected cotinine levels were used to control the different urinary excretion rates in the same manner as for BPA and MDA.

**Table 3:** Genotyped polymorphisms.

Gene	Rs No.	HGVS Name	Chromosome no.	Position	Amino Acid Change	Call Rate (%)	*p* Value_hwe_
*PARP4*	rs12863638	c.273+2528G>T	13	Intron (upstream)	-	99.2	>0.05
	rs3783073	c.879+374T>C	13	Intron	-	98.8	>0.05
	rs2275660	c.2695G>A	13	Codon899	Ala899Thr	98.6	>0.05
*XRCC3*	rs861531	c.406+533G>T	14	Intron	-	99.4	>0.05
	rs12432907	c.561+1132G>A	14	Intron (downstream)	-	100	>0.05
	rs861537	c.562-1162G>A	14	Intron	-	99.6	>0.05
*RAD51*	rs2619681	c.-3+1502T>C	15	Intron (upstream)	-	99.6	>0.05
	rs2304579	c.87+110A>G	15	Intron	-	99.6	<0.05 *
	rs2412547	c.530+1598C>A	15	Intron	-	99.8	>0.05

**Table 4:** The relationships between BPA exposure and oxidative stress by repair gene polymorphisms and combined diplotypes.

Gene	Rs Number	Genotype	Subject No. (%)	Observation No. (%)	β (95% CI)	*p* Value
Total			482 (100)	1416 (100)	0.10 (0.07, 0.13)	<0.0001 *
*PARP4*	rs12863638	GG	216 (45.2)	636 (45.3)	0.08 (0.03, 0.12)	0.0008 *
		GT	220 (46.0)	637 (45.4)	0.12 (0.08, 0.16)	<0.0001 *
		TT	42 (8.8)	131 (9.3)	0.10 (0.01, 0.18)	0.0317 *
		GT+TT	262 (54.8)	768 (54.7)	0.12 (0.08, 0.15)	<0.0001 *
	rs3783073	CC	200 (42.0)	590 (42.2)	0.10 (0.06, 0.14)	<0.0001 *
		CT	223 (46.9)	662 (47.3)	0.10 (0.06, 0.14)	<0.0001 *
		TT	53 (11.1)	147 (10.5)	0.10 (0.03, 0.17)	0.0077 *
		CT+TT	276 (58.0)	809 (57.8)	0.10 (0.06, 0.14)	<0.0001 *
	rs2275660	AA	203 (42.6)	581 (41.7)	0.11 (0.07, 0.15)	<0.0001 *
		AG	211 (44.4)	632 (45.3)	0.10 (0.06, 0.15)	<0.0001 *
		GG	62 (13.0)	181 (13.0)	0.03 (−0.05, 0.10)	0.4598
		AG+GG	273 (57.4)	813 (58.3)	0.08 (0.04, 0.12)	<0.0001 *
	With G-C-G haplotype		246 (52.6)	728 (53.1)	0.08 (0.04, 0.13)	0.0001 *
	Without G-C-G haplotype		222 (47.4)	644 (46.9)	0.11 (0.07, 0.14)	<0.0001 *
*XRCC3*	rs861531	GG	422 (88.1)	1242 (88.2)	0.11 (0.08, 0.14)	<0.0001 *
		GT	56 (11.7)	163 (11.6)	0.02 (−0.08, 0.11)	0.7143
		TT	1 (0.2)	3 (0.2)	-	-
		GT+TT	57 (11.9)	166 (11.8)	0.01 (−0.08, 0.10)	0.8945
	rs12432907	AA	138 (28.6)	411 (29.0)	0.10 (0.06, 0.15)	<0.0001 *
		AG	239 (49.6)	677 (47.8)	0.10 (0.05, 0.14)	<0.0001 *
		GG	105 (21.8)	328 (23.2)	0.10 (0.04, 0.15)	0.0006 *
		AG+GG	344 (71.4)	1005 (71.0)	0.10 (0.06, 0.13)	<0.0001 *
	rs861537	GG	189 (39.4)	563 (39.9)	0.10 (0.06, 0.15)	<0.0001 *
		GA	223 (46.4)	629 (44.5)	0.09 (0.05, 0.14)	<0.0001 *
		AA	68 (14.2)	220 (15.6)	0.09 (0.03, 0.16)	0.0076 *
		GA+AA	291 (60.6)	849 (60.1)	0.09 (0.06, 0.13)	<0.0001 *
	With G-A-G haplotype		374 (78.1)	1080 (76.7)	0.10 (0.07, 0.13)	<0.0001 *
	Without G-A-G haplotype		105 (21.9)	328 (23.3)	0.10 (0.04, 0.15)	0.0006 *
*RAD51*	rs2619681	TT	235 (49.0)	675 (47.9)	0.10 (0.07, 0.14)	<0.0001 *
		TC	213 (44.4)	642 (45.6)	0.09 (0.05, 0.14)	<0.0001 *
		CC	32 (6.6)	92 (6.5)	0.13 (0.01, 0.25)	0.0369 *
		TC+CC	245 (51.0)	734 (52.1)	0.10 (0.06, 0.14)	<0.0001 *
	rs2304579	AA	373 (77.7)	1081 (76.6)	0.10 (0.07, 0.13)	<0.0001 *
		AG	97 (20.2)	299 (21.2)	0.09 (0.04, 0.15)	0.0011 *
		GG	10 (2.1)	32 (2.2)	0.08 (−0.20, 0.36)	0.5706
		AG+GG	107 (22.3)	331 (23.4)	0.09 (0.04, 0.14)	0.0011 *
	rs2412547	AA	334 (69.4)	959 (67.8)	0.09 (0.06, 0.12)	<0.0001 *
		AC	132 (27.5)	409 (28.9)	0.12 (0.07, 0.17)	<0.0001 *
		CC	15 (3.1)	47 (3.3)	0.15 (−0.07, 0.37)	0.1631
		AC+CC	147 (30.6)	456 (32.2)	0.12 (0.07, 0.16)	<0.0001 *
	With T-A-A haplotype		448 (93.5)	1317 (93.5)	0.10 (0.07, 0.12)	<0.0001 *
	Without T-A-A haplotype		31 (6.5)	91 (6.5)	0.13 (0.005, 0.25)	0.0423 *

Each haplotype was composed of rs12863638, rs3783073, and rs2275660 for *PARP4*, rs861531, rs12432907, and rs861537 for *XRCC3*, and rs2619681, rs2304579, and rs2412547 for *RAD51*. Estimated (β) values were obtained after adjustment for age, sex, body mass index (BMI), alcohol consumption status, exercise, cotinine level and PM_10_ on lag day 2, and mean temperature and dewpoint on the day. In the models, creatinine-corrected BPA and MDA levels were used after natural log-transformation.

**Table 5:** The relationships between BPA exposure and liver abnormality by repair gene polymorphisms and combined diplotypes.

Gene	Rs Number	Genotype	Subject No. (%)	Control No. (%)	Case No. (%)	Crude OR (95% CI)	*p* Value	Adjusted OR (95% CI)	*p* Value
Total			499 (100)	970 (96.8)	32 (3.2)	1.39 (1.004, 1.93)	0.0475 *	1.46 (1.04, 2.05)	0.0308 *
*PARP4*	rs12863638	GG	223 (45.1)	428 (44.5)	17 (53.1)	1.42 (0.90, 2.23)	0.1320	1.43 (0.89, 2.31)	0.1367
		GT	227 (45.8)	440 (45.7)	13 (40.6)	1.64 (0.96, 2.81)	0.0703	-	-
		TT	45 (9.1)	94 (9.8)	2 (6.3)	0.61 (0.20, 1.82)	0.3687	-	-
		GT+TT	272 (54.9)	534 (55.5)	15 (46.9)	1.42 (0.87, 2.31)	0.1592	-	-
	rs3783073	CC	205 (41.6)	402 (41.9)	16 (50.0)	1.87 (1.20, 2.90)	0.0056 *	1.96 (1.18, 3.26)	0.0093 *
		CT	234 (47.4)	458 (47.7)	11 (34.4)	1.46 (0.79, 2.68)	0.2229	-	-
		TT	54 (11.0)	100 (10.4)	5 (15.6)	0.69 (0.37, 1.31)	0.2516	0.99 (0.26, 3.79)	0.9887
		CT+TT	288 (58.4)	558 (58.1)	16 (50.0)	1.02 (0.65, 1.61)	0.9213	1.07 (0.66, 1.72)	0.7968
	rs2275660	AA	214 (43.5)	415 (43.55)	12 (38.7)	0.84 (0.53, 1.33)	0.4463	0.93 (0.54, 1.61)	0.8053
		AG	214 (43.5)	415 (43.55)	10 (32.3)	1.89 (1.05, 3.41)	0.0338 *	-	-
		GG	64 (13.0)	123 (12.9)	9 (29.0)	2.45 (1.24, 4.83)	0.0106 *	2.63 (1.07, 6.44)	0.0354 *
		AG+GG	278 (56.5)	538 (56.45)	19 (61.3)	2.12 (1.34, 3.25)	0.0006 *	2.14 (1.35, 3.39)	0.0014 *
	With G-C-G haplotype		251 (51.9)	485 (51.65)	19 (61.3)	2.14 (1.39, 3.28)	0.0006 *	2.16 (1.35, 3.45)	0.0014 *
	Without G-C-G haplotype		233 (48.1)	454 (48.35)	12 (38.7)	0.83 (0.53, 1.32)	0.4378	0.92 (0.54, 1.59)	0.7702
*XRCC3*	rs861531	GG	435 (87.7)	842 (87.3)	32 (100)	1.40 (1.01, 1.94)	0.0454 *	1.48 (1.05, 2.09)	0.0250 *
		GT	59 (11.9)	120 (12.4)	0 (0)	-	-	-	-
		TT	2 (0.4)	3 (0.3)	0 (0)	-	-	-	-
		GT+TT	61 (12.3)	123 (12.7)	0 (0)	-	-	-	-
	rs12432907	AA	139 (27.9)	272 (28.0)	9 (28.1)	1.03 (0.58, 1.81)	0.9324	1.08 (0.62, 1.89)	0.7870
		AG	251 (50.3)	468 (48.3)	13 (40.6)	1.86 (1.16, 3.00)	0.0107 *	2.17 (1.24, 3.78)	0.0065 *
		GG	109 (21.8)	230 (23.7)	10 (31.3)	1.24 (0.64, 2.41)	0.5280	-	-
		AG+GG	360 (72.1)	698 (72.0)	23 (71.9)	1.56 (1.06, 2.30)	0.0250 *	1.70 (1.11, 2.61)	0.0155 *
	rs861537	GG	193 (38.8)	380 (39.3)	12 (37.5)	1.29 (0.77, 2.18)	0.3311	1.31 (0.78, 2.21)	0.3081
		GA	232 (46.7)	434 (44.8)	12 (37.5)	1.54 (0.93, 2.55)	0.0939	1.80 (1.02, 3.20)	0.0434 *
		AA	72 (14.5)	154 (15.9)	8 (25.0)	1.43 (0.63, 3.23)	0.3846	-	-
		GA+AA	304 (61.2)	588 (60.7)	20 (62.5)	1.46 (0.96, 2.22)	0.0776	1.60 (1.00, 2.57)	0.0524
	With G-A-G haplotype		387 (78.0)	735 (76.2)	22 (68.75)	1.49 (1.02, 2.17)	0.0400 *	1.57 (1.06, 2.32)	0.0249 *
	Without G-A-G haplotype		109 (22.0)	230 (23.8)	10 (31.25)	1.24 (0.64, 2.41)	0.5280	-	-
*RAD51*	rs2619681	TT	244 (49.1)	464 (48.0)	19 (59.4)	1.71 (1.10, 2.66)	0.0186 *	-	-
		TC	217 (43.7)	423 (43.8)	12 (37.5)	0.96 (0.60, 1.53)	0.8467	0.95 (0.58, 1.56)	0.8353
		CC	36 (7.2)	79 (8.2)	1 (3.1)	9.18 (0.27, 314.8)	0.2122	-	-
		TC+CC	253 (50.9)	502 (52.0)	13 (40.6)	1.03 (0.64, 1.66)	0.9141	1.00 (0.61, 1.62)	0.9860
	rs2304579	AA	386 (77.7)	737 (76.1)	28 (87.5)	1.62 (1.14, 2.30)	0.0079 *	1.65 (1.14, 2.40)	0.0083 *
		AG	99 (19.9)	202 (20.9)	4 (12.5)	0.63 (0.34, 1.15)	0.1323	-	-
		GG	12 (2.4)	29 (3.0)	0 (0)	-	-	-	-
		AG+GG	111 (22.3)	231 (23.9)	4 (12.5)	0.62 (0.33, 1.15)	0.1247	-	-
	rs2412547	AA	345 (69.3)	657 (67.8)	23 (71.9)	1.70 (1.16, 2.50)	0.0072 *	1.79 (1.18, 2.71)	0.0067 *
		AC	136 (27.3)	274 (28.3)	8 (25.0)	0.73 (0.44, 1.21)	0.2174	0.74 (0.41, 1.36)	0.3311
		CC	17 (3.4)	38 (3.9)	1 (3.1)	111.3 (0.01, >999)	0.2926	-	-
		AC+CC	153 (30.7)	312 (32.2)	9 (28.1)	0.80 (0.47, 1.35)	0.4032	0.81 (0.46, 1.43)	0.4727
	With T-A-A haplotype		461 (92.9)	887 (91.9)	31 (96.9)	1.35 (0.97, 1.87)	0.0712	1.43 (1.01, 2.01)	0.0422 *
	Without T-A-A haplotype		35 (7.1)	78 (8.1)	1 (3.1)	9.13 (0.26, 315.4)	0.2140	-	-

Each haplotype was composed of rs12863638, rs3783073, and rs2275660 for *PARP4*, rs861531, rs12432907, and rs861537 for *XRCC3*, and rs2619681, rs2304579, and rs2412547 for *RAD51*. Adjusted ORs were obtained after adjustment for age, sex, BMI, alcohol consumption status, exercise, cotinine levels, and LDL-C. In the models, creatinine-corrected BPA levels were used after natural log-transformation.

**Table 6:** The effects of repair gene polymorphisms and combined diplotypes on oxidative stress.

Gene	Rs Number	Genotype	Subject No. (%)	Observation No. (%)	β ^1^ (95% CI)	*p* Value ^1^	β ^2^ (95% CI)	*p* Value ^2^	*p* Value ^3^
*PARP4*	rs12863638	GG	216 (45.2)	636 (45.3)	0.00 (ref.)	0.5053	0.00 (ref.)	0.5826	0.2745
		GT	220 (46.0)	637 (45.4)	−0.005 (−0.08, 0.07)		−0.01 (−0.08, 0.06)		
		TT	42 (8.8)	131 (9.3)	0.07 (−0.06, 0.20)		0.06 (−0.07, 0.18)		
		GT+TT	262 (54.8)	768 (54.7)	0.01 (−0.06, 0.08)	0.8282	0.001 (−0.07, 0.07)	0.9711	0.1642
	rs3783073	CC	200 (42.0)	590 (42.2)	0.00 (ref.)	0.7429	0.00 (ref.)	0.6755	0.9294
		CT	223 (46.9)	662 (47.3)	0.03 (−0.05, 0.10)		0.03 (−0.05, 0.11)		
		TT	53 (11.1)	147 (10.5)	0.03 (−0.09, 0.15)		0.04 (−0.08, 0.16)		
		CT+TT	276 (58.0)	809 (57.8)	0.03 (−0.04, 0.10)	0.4411	0.03 (−0.04, 0.10)	0.3849	0.8805
	rs2275660	AA	203 (42.6)	581 (41.7)	0.00 (ref.)	0.7212	0.00 (ref.)	0.8744	0.1704
		AG	211 (44.4)	632 (45.3)	0.02 (−0.05, 0.10)		0.02 (−0.06, 0.09)		
		GG	62 (13.0)	181 (13.0)	−0.02 (−0.13, 0.09)		−0.01 (−0.12, 0.10)		
		AG+GG	273 (57.4)	813 (58.3)	0.01 (−0.06, 0.09)	0.6973	0.01 (−0.06, 0.08)	0.7708	0.2956
	With G-C-G haplotype		246 (52.6)	728 (53.1)	0.00 (ref.)	0.5984	0.00 (ref.)	0.6258	0.4182
	Without G-C-G haplotype		222 (47.4)	644 (46.9)	0.02 (-0.05, 0.09)		0.02 (−0.05, 0.09)		
*XRCC3*	rs861531	GG	422 (88.1)	1242 (88.2)	0.00 (ref.)	0.7101	0.00 (ref.)	0.4359	0.0445 *
		GT	56 (11.7)	163 (11.6)	0.03 (−0.08, 0.14)		0.03 (−0.08, 0.14)		
		TT	1 (0.2)	3 (0.2)	0.22 (−0.53, 0.98)		0.47 (−0.30, 1.23)		
		GT+TT	57 (11.9)	166 (11.8)	0.04 (−0.07, 0.15)	0.5054	0.04 (−0.07, 0.15)	0.5214	0.0077 *
	rs12432907	AA	138 (28.6)	411 (29.0)	0.00 (ref.)	0.7425	0.00 (ref.)	0.8727	0.9978
		AG	239 (49.6)	677 (47.8)	0.03 (−0.05, 0.11)		0.02 (−0.06, 0.10)		
		GG	105 (21.8)	328 (23.2)	0.01 (−0.09, 0.10)		0.01 (−0.09, 0.10)		
		AG+GG	344 (71.4)	1005 (71.0)	0.02 (−0.06, 0.10)	0.5772	0.02 (−0.06, 0.09)	0.6821	0.9620
	rs861537	GG	189 (39.4)	563 (39.9)	0.00 (ref.)	0.6894	0.00 (ref.)	0.7076	0.9619
		GA	223 (46.4)	629 (44.5)	0.01 (−0.06, 0.09)		0.02 (−0.06, 0.09)		
		AA	68 (14.2)	220 (15.6)	-0.03 (−0.14, 0.07)		−0.03 (−0.13, 0.08)		
		GA+AA	291 (60.6)	849 (60.1)	0.001 (−0.07, 0.07)	0.9783	0.01 (−0.07, 0.08)	0.8847	0.8076
	With G-A-G haplotype		374 (78.1)	1080 (76.7)	0.00 (ref.)	0.7569	0.00 (ref.)	0.8645	0.9786
	Without G-A-G haplotype		105 (21.9)	328 (23.3)	−0.01 (−0.10, 0.07)		−0.01 (−0.09, 0.07)		
*RAD51*	rs2619681	TT	235 (49.0)	675 (47.9)	0.00 (ref.)	0.3768	0.00 (ref.)	0.3476	0.7963
		TC	213 (44.4)	642 (45.6)	0.03 (−0.04, 0.10)		0.02 (−0.05, 0.09)		
		CC	32 (6.6)	92 (6.5)	−0.07 (−0.21, 0.08)		−0.09 (−0.23, 0.06)		
		TC+CC	245 (51.0)	734 (52.1)	0.02 (−0.05, 0.09)	0.6532	0.01 (−0.06, 0.08)	0.8429	0.7429
	rs2304579	AA	373 (77.7)	1081 (76.6)	0.00 (ref.)	0.9218	0.00 (ref.)	0.9218	0.8027
		AG	97 (20.2)	299 (21.2)	−0.02 (−0.10, 0.07)		−0.01 (−0.10, 0.07)		
		GG	10 (2.1)	32 (2.2)	−0.002 (−0.23, 0.23)		0.03 (−0.21, 0.26)		
		AG+GG	107 (22.3)	331 (23.4)	−0.02 (−0.10, 0.07)	0.7025	−0.01 (−0.09, 0.07)	0.8057	0.5044
	rs2412547	AA	334 (69.4)	959 (67.8)	0.00 (ref.)	0.2066	0.00 (ref.)	0.2168	0.8329
		AC	132 (27.5)	409 (28.9)	0.07 (−0.01, 0.15)		0.07 (-0.01, 0.15)		
		CC	15 (3.1)	47 (3.3)	0.004 (−0.19, 0.20)		−0.02 (−0.21, 0.18)		
		AC+CC	147 (30.6)	456 (32.2)	0.06 (−0.01, 0.14)	0.0975	0.06 (−0.02, 0.13)	0.1213	0.6023
	With T-A-A haplotype		448 (93.5)	1317 (93.5)	0.00 (ref.)	0.2697	0.00 (ref.)	0.1977	0.6292
	Without T-A-A haplotype		31 (6.5)	91 (6.5)	−0.08 (−0.22, 0.06)		−0.09 (−0.24, 0.05)		

Each haplotype was composed of rs12863638, rs3783073, and rs2275660 for *PARP4*, rs861531, rs12432907, and rs861537 for *XRCC3*, and rs2619681, rs2304579, and rs2412547 for *RAD51*. ^1^Estimated (β) and ^1^*p* values were obtained after adjustment for age, sex, BMI, alcohol consumption status, exercise, cotinine level and PM_10_ on lag day 2, and mean temperature and dewpoint on the day. ^2^Estimated (β) and ^2^*p* values were obtained after adjustment for age, sex, BMI, alcohol consumption status, exercise, cotinine level and PM_10_ on lag day 2, mean temperature and dewpoint on the day, and BPA level. A ^3^*p* value was obtained for the interaction between each polymorphism and BPA level. In the models, creatinine-corrected BPA and MDA levels were used after natural log-transformation.

**Table 7:** The effects of repair gene polymorphisms and combined diplotypes on liver abnormality.

Gene	Rs Number	Genotype	Subject No. (%)	Control No. (%)	Case No. (%)	Crude OR (95% CI)	*p* Value	Adjusted OR ^1^ (95% CI)	*p* Value ^1^	Adjusted OR ^2^ (95% CI)	*p* Value ^2^	*p* Value ^3^
*PARP4*	rs12863638	GG	223 (45.1)	428 (44.5)	17 (53.1)	1.0 (ref.)	0.6055	1.0 (ref.)	0.6454	1.0 (ref.)	0.5581	0.2711
		GT	227 (45.8)	440 (45.7)	13 (40.6)	0.7 (0.34, 1.60)		0.8 (0.35, 1.72)		0.7 (0.32, 1.63)		
		TT	45 (9.1)	94 (9.8)	2 (6.3)	0.5 (0.12, 2.51)		0.5 (0.11, 2.53)		0.5 (0.10, 2.37)		
		GT+TT	272 (54.9)	534 (55.5)	15 (46.9)	0.7 (0.33, 1.48)	0.3498	0.7 (0.34, 1.57)	0.4168	0.7 (0.31, 1.48)	0.3277	0.9662
	rs3783073	CC	205 (41.6)	402 (41.9)	16 (50.0)	1.0 (ref.)	0.3424	1.0 (ref.)	0.3799	1.0 (ref.)	0.3839	0.0936
		CT	234 (47.4)	458 (47.7)	11 (34.4)	0.6 (0.27, 1.36)		0.6 (0.25, 1.31)		0.6 (0.25, 1.37)		
		TT	54 (11.0)	100 (10.4)	5 (15.6)	1.2 (0.41, 3.71)		1.0 (0.29, 3.38)		1.1 (0.33, 3.89)		
		CT+TT	288 (58.4)	558 (58.1)	16 (50.0)	0.7 (0.34, 1.50)	0.3748	0.6 (0.29, 1.39)	0.2556	0.7 (0.31, 1.47)	0.3217	0.1174
	rs2275660	AA	214 (43.5)	415 (43.55)	12 (38.7)	1.0 (ref.)	0.0651	1.0 (ref.)	0.0387 *	1.0 (ref.)	0.0387 *	0.0289 *
		AG	214 (43.5)	415 (43.55)	10 (32.3)	0.9 (0.35, 2.07)		1.0 (0.38, 2.50)		0.9 (0.37, 2.44)		
		GG	64 (13.0)	123 (12.9)	9 (29.0)	2.6 (0.99, 6.73)		3.3 (1.16, 9.13)		3.3 (1.16, 9.27)		
		AG+GG	278 (56.5)	538 (56.45)	19 (61.3)	1.2 (0.57, 2.69)	0.5838	1.5 (0.64, 3.36)	0.3707	1.4 (0.62, 3.30)	0.4030	0.0085 *
	With G-C-G haplotype		251 (51.9)	485 (51.65)	19 (61.3)	1.0 (ref.)	0.3030	1.0 (ref.)	0.1832	1.0 (ref.)	0.1968	0.0078 *
	Without G-C-G haplotype		233 (48.1)	454 (48.35)	12 (38.7)	0.7 (0.31, 1.44)		0.6 (0.25, 1.31)		0.6 (0.25, 1.33)		
*XRCC3*	rs861531	GG	435 (87.7)	842 (87.3)	32 (100)	1.0 (ref.)	-	1.0 (ref.)	-	1.0 (ref.)	-	-
		GT	59 (11.9)	120 (12.4)	0 (0)	-		-		-		
		TT	2 (0.4)	3 (0.3)	0 (0)	-		-		-		
		GT+TT	61 (12.3)	123 (12.7)	0 (0)	-	-	-	-	-	-	-
	rs12432907	AA	139 (27.9)	272 (28.0)	9 (28.1)	1.0 (ref.)	0.6528	1.0 (ref.)	0.8071	1.0 (ref.)	0.7039	0.3364
		AG	251 (50.3)	468 (48.3)	13 (40.6)	0.8 (0.34, 2.05)		0.8 (0.32, 2.06)		0.7 (0.28, 1.84)		
		GG	109 (21.8)	230 (23.7)	10 (31.3)	1.3 (0.48, 3.34)		1.1 (0.40, 3.07)		1.0 (0.37, 2.87)		
		AG+GG	360 (72.1)	698 (72.0)	23 (71.9)	1.0 (0.43, 2.22)	0.9529	0.9 (0.39, 2.13)	0.8293	0.8 (0.35, 1.93)	0.6547	0.2616
	rs861537	GG	193 (38.8)	380 (39.3)	12 (37.5)	1.0 (ref.)	0.4748	1.0 (ref.)	0.6446	1.0 (ref.)	0.6306	0.9193
		GA	232 (46.7)	434 (44.8)	12 (37.5)	0.9 (0.38, 2.09)		0.8 (0.35, 2.01)		0.8 (0.33, 1.94)		
		AA	72 (14.5)	154 (15.9)	8 (25.0)	1.6 (0.61, 4.28)		1.4 (0.48, 3.92)		1.3 (0.46, 3.84)		
		GA+AA	304 (61.2)	588 (60.7)	20 (62.5)	1.1 (0.51, 2.33)	0.8363	1.0 (0.44, 2.17)	0.9511	0.9 (0.42, 2.10)	0.8783	0.7293
	With G-A-G haplotype		387 (78.0)	735 (76.2)	22 (68.75)	1.0 (ref.)	0.4077	1.0 (ref.)	0.6192	1.0 (ref.)	0.6124	0.6686
	Without G-A-G haplotype		109 (22.0)	230 (23.8)	10 (31.25)	1.4 (0.63, 3.16)		1.2 (0.53, 2.94)		1.3 (0.53, 2.97)		
*RAD51*	rs2619681	TT	244 (49.1)	464 (48.0)	19 (59.4)	1.0 (ref.)	0.4356	1.0 (ref.)	0.5784	1.0 (ref.)	0.5669	0.0965
		TC	217 (43.7)	423 (43.8)	12 (37.5)	0.7 (0.33, 1.55)		0.8 (0.35, 1.72)		0.8 (0.34, 1.69)		
		CC	36 (7.2)	79 (8.2)	1 (3.1)	0.3 (0.04, 2.52)		0.3 (0.05, 3.05)		0.4 (0.05, 3.08)		
		TC+CC	253 (50.9)	502 (52.0)	13 (40.6)	0.7 (0.31, 1.38)	0.2667	0.7 (0.33, 1.55)	0.3920	0.7 (0.32, 1.53)	0.3732	0.0867
	rs2304579	AA	386 (77.7)	737 (76.1)	28 (87.5)	1.0 (ref.)	-	1.0 (ref.)	-	1.0 (ref.)	-	-
		AG	99 (19.9)	202 (20.9)	4 (12.5)	-		-		-		
		GG	12 (2.4)	29 (3.0)	0 (0)	-		-		-		
		AG+GG	111 (22.3)	231 (23.9)	4 (12.5)	0.5 (0.16, 1.41)	0.1784	0.5 (0.16, 1.48)	0.2023	0.5 (0.16, 1.56)	0.2357	0.0122 *
	rs2412547	AA	345 (69.3)	657 (67.8)	23 (71.9)	1.0 (ref.)	0.9169	1.0 (ref.)	0.9433	1.0 (ref.)	0.9634	0.0241 *
		AC	136 (27.3)	274 (28.3)	8 (25.0)	0.9 (0.37, 2.02)		0.9 (0.36, 2.11)		0.9 (0.37, 2.19)		
		CC	17 (3.4)	38 (3.9)	1 (3.1)	0.8 (0.09, 6.25)		0.8 (0.09, 7.20)		0.8 (0.09, 7.53)		
		AC+CC	153 (30.7)	312 (32.2)	9 (28.1)	0.8 (0.37, 1.92)	0.6879	0.9 (0.37, 2.01)	0.7354	0.9 (0.38, 2.09)	0.7899	0.0229 *
	With T-A-A haplotype		461 (92.9)	887 (91.9)	31 (96.9)	1.0 (ref.)	0.3425	1.0 (ref.)	0.4155	1.0 (ref.)	0.4193	0.3130
	Without T-A-A haplotype		35 (7.1)	78 (8.1)	1 (3.1)	0.4 (0.05, 2.88)		0.4 (0.05, 3.37)		0.4 (0.05, 3.42)		

**Each haplotype was composed of rs12863638, rs3783073, and rs2275660 for *PARP4*, rs861531, rs12432907, and rs861537 for *XRCC3*, and rs2619681, rs2304579, and rs2412547 for *RAD51*.**^1^ Adjusted OR and ^1^*p* values were obtained after adjustment for age, sex, BMI, alcohol consumption status, exercise, cotinine level, and LDL-C level. ^2^ Adjusted OR and ^2^*p* values were obtained after adjustment for age, sex, BMI, alcohol consumption status, exercise, cotinine level, LDL-C level, and BPA level. A ^3^ *p* value was obtained for the interaction between each polymorphism and BPA level. In the models, creatinine-corrected BPA were used after natural log-transformation.
